# Insight into Sulfur‐Containing Zwitter‐Molecule Boosting Zn Anode: from Electrolytes to Electrodes

**DOI:** 10.1002/advs.202400094

**Published:** 2024-02-23

**Authors:** Weihao Song, Jiaxing Liu, Shengpu Rao, Ming Zhao, Yanqun Lv, Shunshun Zhao, Qing Ma, Bing Wu, Chengjin Zheng, Shimou Chen, Zhilin Li, Jin Niu, Feng Wang

**Affiliations:** ^1^ State Key Laboratory of Chemical Resource Engineering Laboratory of Electrochemical Process and Technology for materials Beijing University of Chemical Technology Beijing 100029 P. R. China; ^2^ Beijing Advanced Innovation Center for Soft Matter Science and Engineering Beijing University of Chemical Technology Beijing 100029 P. R. China

**Keywords:** electrolytes additive, practical performance, sulfur‐containing functional group, Zn anode, zwitter molecule

## Abstract

Numerous organic electrolytes additives have been reported to improve Zn anode performance in aqueous Zn metal batteries (AZMBs). However, the modification mechanism needs to be further revealed in consideration of different environments for electrolytes and electrodes during the charge‐discharge process. Herein, sulfur‐containing zwitter‐molecule (methionine, Met) is used as an additive for ZnSO_4_ electrolytes. In electrolytes, Met reduces the H_2_O coordination number and facilitates the desolvation process by virtue of functional groups (─COOH, ─NH_2_, C─S─C), accelerating Zn^2+^ transference kinetics and decreasing the amount of active water. On electrodes, Met prefers to adsorb on Zn (002) plane and further transforms into a zincophilic protective layer containing C─SO_x_─C through an in situ electrochemical oxidization, suppressing H_2_ evolution/corrosion reactions and guiding dendrite‐free Zn deposition. By using Met‐containing ZnSO_4_ electrolytes, the Zn//Zn cells show superior cycling performance under 30 mA cm^−2^/30 mA h cm^−2^. Moreover, the full cells Zn//NH_4_V_4_O_10_ full cells using the modified electrolytes exhibit good performance at temperatures from −8 to 60 °C. Notably, a high energy density of 105.30 W h kg^−1^ can be delivered using a low N/P ratio of 1.2, showing a promising prospect of Met electrolytes additives for practical use.

## Graphical abstract

Sulfur‐containing zwitter‐molecule methionine (Met) is used as an additive for the ZnSO_4_ electrolytes to modify the Zn anode, resulting in superior half‐cell and full‐cell performance even under extreme test conditions. Detailed experimental analysis and theoretical calculation results revealed the different roles of Met played from electrolytes to electrodes.

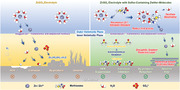

## 1 Introduction

Aqueous Zn metal batteries (AZMBs) are receiving more and more attentions due to their eco‐friendliness, high capacity, and good safety.^[^ ^1^ ^]^ Metallic Zn has advantages of low potential (−0.76 V vs SHE),^[^ ^2^ ^]^ high specific capacity (820 mA h g^−1^ or 5855 mA h cm^−3^), and modest reactivity with water.^[^ ^3^ ^]^ Therefore, Zn anodes are commonly used to build high‐energy AZMBs.^[^ ^4^ ^]^ However, there are still some serious issues for Zn anodes, which hinder their practical applications.^[^ ^5^ ^]^

**Scheme 1:**
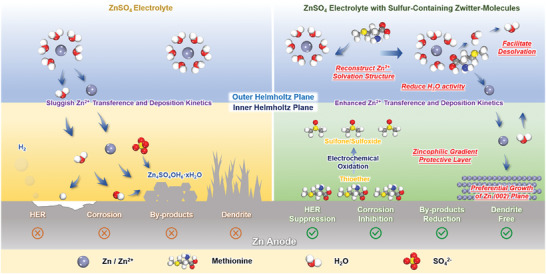
Schematic illustration of Zn plating behaviors in ZSO (left) and Met‐ZSO (right).

As shown in **Scheme** **1**, there are two planes near the Zn anode within the electrolytes: outer Helmholtz plane and inner Helmholtz plane. For conventional ZnSO_4_ electrolytes (ZSO), Zn^2+^ in the formation of (Zn(OH_2_)_6_)^2+^ enter into the outer Helmholtz plane from diffusion layer during the charging process, further decoupling with H_2_O molecules by desolvation process (Scheme 1, left). However, the strong interaction between Zn^2+^ and H_2_O causes a high energy barrier for desolvation, leading to a sluggish Zn^2+^ transference and deposition kinetics.^[^ ^6^ ^]^ Meanwhile, if H_2_O molecules are not removed from the solvation sheath, the coordinated H_2_O with high activity will easily form H_2_ and OH^−^ on Zn anode within the inner Helmholtz plane. The H_2_ evolution reaction (HER) would increase the gas pressure, resulting in swelling or even rupture of the AZMBs. Additionally, Zn anode suffers from anodic corrosion and transforms into soluble Zn^2+^, which then partially reacts with OH^−^ and SO_4_^2−^ to form Zn_4_SO_4_OH_6_·xH_2_O.^[^ ^7^ ^]^ These byproducts with loose structures cannot stop further corrosion reactions and the persistent side reactions can result in continuous electrolytes consumption, low Coulombic efficiency (CE), and poor cyclability. The accumulated byproducts can also increase the electrodes polarization, which probably induces dendrite growth due to the heterogeneous electric field distribution, thus increasing the risk of internal short‐circuiting.^[^ ^8^ ^]^

Many strategies have been proposed to address the above issues for Zn anodes, including HER, corrosion, by‐products, and dendrite. These reported methods could be divided into two categories: anode design and electrolytes engineering.^[^ ^9^, ^10^ ^]^ The former primarily includes Zn alloy,^[^ ^11^ ^]^ surface modification,^[^ ^12^ ^]^ and host utilization.^[^ ^13^ ^]^ However, anode design commonly suffers from complex procedures for material synthesis and unsatisfying anode performance, especially under high capacity and current. Electrolytes engineering (e.g., building eutectic electrolytess, using electrolytes additives, employing solid electrolytess) can fundamentally improve the Zn anode performance with high efficiency.^[^ ^14^ ^]^ Recently, organic electrolytes additives have received much attention due to their low costs and good effects for Zn anode modification.^[^ ^15^ ^]^ It is widely recognized that organic additives with specific functional groups regulate Zn^2+^ solvation structure in electrolytes and form a protective layer on the Zn anode,^[^ ^16^, ^17^ ^]^ thus suppressing HER/corrosion reaction and alleviating by‐product/dendrite formation.^[^ ^18^, ^19^, ^20^ ^]^ However, the dynamic change of organic additives from electrolytes to Zn anode is not fully understood. Specifically, it is easily neglected that the structural compositions of organic additives might change due to the different environments in electrolytes and on the Zn anode during the charge‐discharge process, which would lead to a solid electrolytes interface (SEI) with different functionalities, further affecting the performance of Zn anode. In addition to the modification mechanism, researchers should also pay more attention to the real performance of Zn anodes and AZMBs under extreme test conditions (including large depth of discharge, low N/P ratio, and wide temperature), which are the key to the practical applications of AZMBs in future.

In this work, we used a sulfur‐containing zwitter‐molecule, methionine (Met), as the electrolytes additive for AZMBs. Detailed experimental analysis and theoretical calculation results revealed the different roles of Met played from electrolytes to electrodes (Scheme 1, right). In Met‐containing ZSO (Met‐ZSO), Met not only reduced the activities of H_2_O molecules due to carboxyl (─COOH) but also facilitated the desolvation process by virtue of amino (─NH_2_) and thioether (C─S─C), thus enhancing Zn^2+^ transport and suppressing HER. On the Zn anode, oxidized Met with sulfoxide or sulfone (C─SO_x_─C) preferentially adsorbed on the Zn (002) plane and formed a zincophilic gradient protective layer, inhibiting corrosion reactions and guiding dendrite‐free Zn deposition. As a result, Met‐ZSO enabled superior half‐cell and full‐cell performance even under extreme test conditions, showing good practicability of Met‐ZSO for AZMBs.

## 2 Results and Discussion

**Figure 1:**
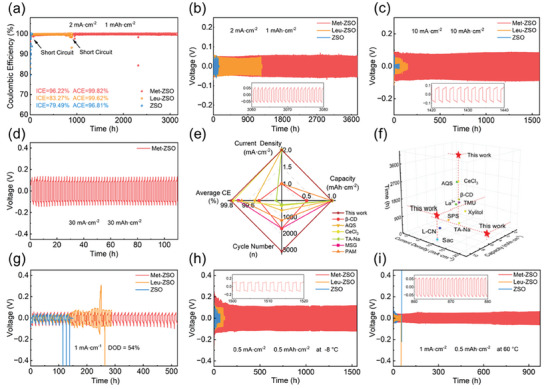
a) CE of the Zn//Cu cells in different electrolytess under 2 mA cm^−2^ with 1 mA h cm^−2^. Cycling performance of the Zn//Zn cells in different electrolytess under b) 2 mA cm^−2^ with 1 mA h cm^−2^, c) 10 mA cm^−2^ with 10 mA h cm^−2^, d) 30 mA cm^−2^ with 30 mA h cm^−2^. Comparison of the performance for e) Zn//Cu cells and f) Zn//Zn cells using Met‐ZSO with recent literature studies. Cycling performance of the Zn//Zn cells in different electrolytess under g) 1 mA cm^−2^ with 5 mA h cm^−2^ at 54% DOD, h) 0.5 mA cm^−2^ with 0.5 mA h cm^−2^ at −8 °C, and i) 1 mA cm^−2^ with 0.5 mA h cm^−2^ at 60 °C.

As one of the biomass derivatives within agricultural products, Met contains three functional groups, including ─COOH, ─NH_2_, and C─S─C (Figure S1, left, Supporting Information). Due to the hydrophilic functional groups, Met could be dissolved in 2 m ZnSO_4_ aqueous solutions and used as additives for ZSO. In order to explore the role of C─S─C, leucine (Leu) was also used as an additives for ZSO, which has similar molecular structure to Met but without C─S─C (Figure S1, right, Supporting Information). Leu‐containing ZSO (Leu‐ZSO) and Met‐ZSO with the same concentration of 10 mm were first evaluated in the Zn//Cu cells at 2 mA cm^−2^/1 mA h cm^−2^. As shown in **Figures** **1**a and S2 (Supporting Information), the Zn//Cu cell using Met‐ZSO exhibited good stability even after 3150 h with a high initial CE of 96.22% and average CE of 99.82%, much longer and higher than those using ZSO and Leu‐ZSO. Moreover, the cell cycled in Met‐ZSO could display a high average CE of 99.32% for 210 cycles even under 10 mA cm^−2^/5 mA h cm^−2^. (Figure S3, Supporting Information). In Zn//Zn cells, Met‐ZSO also enabled the symmetric cells to show excellent cycling performance using moderate plating/deposition conditions with optimized Met concentration (Figure S4, Supporting Information) It should be noted that the Zn//Zn cell using Met‐ZSO had an ultralong lifetime of 3750 h under 2 mA cm^−2^/1 mA h cm^−2^, whereas the cells with ZSO and Leu‐ZSO were short‐circuited after 100 and 1200 h (Figure 1b). Even under harsh conditions of 10 mA cm^−2^/10 mA h cm^−2^ and 20 mA cm^−2^/5 mA h cm^−2^, the Zn//Zn cells with Met‐ZSO still worked stably for 1600 and 620 h, respectively (Figure 1c; Figure S5, Supporting Information). When an ultrahigh current density of 30 mA cm^−2^ was used, the cell using Met‐ZSO could still run properly under a plating/stripping capacity of 1 mA h cm^−2^ or even 30 mA h cm^−2^ (Figure 1d; Figure S6, Supporting Information). The remarkable performance for Met‐ZSO is superior to many recently reported ZSO with other additives (Figure 1e,f; Tables S1 and S2, Supporting Information).^[^ ^21^, ^22^, ^23^, ^24^, ^25^, ^26^, ^27^, ^28^, ^29^, ^30^, ^31^, ^32^ ^]^

Considering about the limited Zn required by the anodes for practical full cells, the Zn//Zn cell with Met‐ZSO was also evaluated under 1 mA cm^−2^/5 mA h cm^−2^ at 54% depth‐of‐discharge (DOD). Satisfyingly, the cell with Met‐ZSO could cycle as long as 520 h without a short circuit, better than those with ZSO and Leu‐ZSO (Figure 1g). Since wide‐temperature performance is also important for the practical application of AZMBs, the Zn//Zn cells were further tested at −8 and 60 °C. Although the cell using Met‐ZSO showed a relatively high voltage hysteresis in the initial cycles, the overpotential quickly stabilized for 1600 h at −8 °C (Figure 1h). However, the comparative cells were short‐circuited only in 150 h because of dendrite formation. At a high temperature of 60 °C, the cell using Met‐ZSO could also exhibit a long‐term cycling performance of 975 h (Figure 1i). On the contrary, a sudden voltage floating occurred for the cells with ZSO and Leu‐ZSO at the initial stage due to the severe HER‐induced cell explosion.^[^ ^33^ ^]^

**Figure 2:**
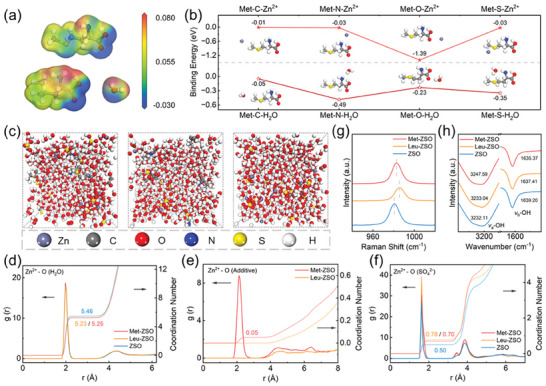
a) ESP distribution of Met, Leu, and H_2_O. b) The calculated binding energy of Met‐Zn^2+^ and Met‐H_2_O with different groups (C: ─CH_3_, O: ─COOH, N: ─NH_2_, S: C─S─C). c) MD simulation snapshots of ZSO, Leu‐ZSO, and Met‐ZSO (from left to right). RDFs for (d) Zn^2+^─O (H_2_O), (e) Zn^2+^─O (additive), and f) Zn^2+^─O (SO_4_^2−^) collected from MD simulations. g) Raman spectra and h) FTIR spectra of different electrolytess.

In order to investigate the modification mechanism of Met and Leu additives, DFT calculations were used to determine the interactions between Zn^2+^ and zwitterionic molecules in electrolytess. **Figure** **2**a indicates that the ─COOH functional groups of Met and Leu have negative electrostatic potential (ESP), thus enhancing the electrostatic attraction between Leu/Met and Zn^2+^. As shown in Figure 2b, ─COOH of Met has the highest binding energy with Zn^2+^ (−1.39 eV) among all the groups, which is also higher than that between ─COOH and H_2_O (−0.23 eV) and that between Zn^2+^ and H_2_O (−0.03 eV, Figure S7, Supporting Information), indicating that Zn^2+^ is preferred to be captured by ─COOH of Met instead of H_2_O. This result also implies that Met additives might lead to the reorganization of Zn^2+^ solvation structure. It is worth mentioning that the C─S─C and ─NH_2_ functional groups have stronger adsorption ability with H_2_O than ─COOH, suggesting that C─S─C and ‐NH_2_ play important role in facilitating desolvation of Zn^2+^ and suppressing activity of H_2_O. Similarly, the calculation results also show that Leu might also affect the solvation structure of Zn^2+^ due to its functional groups of ─COOH and ─NH_2_ (Figure S7, Supporting Information).

MD simulations further demonstrate that Leu and Met have different effects on regulating the solvation structures of Zn^2+^ (Figure 2c). Detailed statistical results of radial distribution functions (RDFs) for Zn^2+^─O (H_2_O), Zn^2+^─O (additive), and Zn^2+^─O (SO_4_^2−^) are displayed in Figure 2d,f, respectively. The average coordination numbers of Zn^2+^─O (H_2_O) in the first‐hydration‐layer of ZSO decrease after the addition of Met and Leu. The lower coordination number is conducive to easier Zn^2+^ desolvation and better transport/deposition kinetics. The average coordination numbers of Zn^2+^─O (Met) and Zn^2+^─S (Met) in the first‐hydration‐layer are 0.05 and 0, respectively (Figure S8, Supporting Information). This suggests that ─COOH enables Met to enter the solvation shell structure and coordinate with Zn^2+^ while C─S─C cannot, which is in accordance with the DFT calculation results. Different from Met, Leu cannot participate into the regulation of the first‐hydration‐layer, which may be due to the steric hindrance of Leu molecule, which is more reliable than the above DFT calculation result.^[^ ^16^ ^]^ Moreover, the average coordination number of Zn^2+^─O (SO_4_^2−^) in the first‐hydration‐layer of Leu‐ZSO is the highest among all the electrolytess, suggesting that Leu causes more SO_4_^2‐^ to coordinate with Zn^2+^. This result was also confirmed by Raman spectra for all the electrolytess (Figure 2g). Leu‐ZSO had the most obvious shift of v(SO_4_^2−^) vibration among all the electrolytess, indicative of the strongest interactions between SO_4_^2−^ and Zn^2+^. Although Leu decreases the coordination number of H_2_O with Zn^2+^, the increased coordination number of SO_4_^2−^ with Zn^2+^ would result in more corrosion by‐products (e.g., Zn_4_SO_4_OH_6_·xH_2_O).^[^ ^34^ ^]^

The effects of the additives on hydrogen bonds of H_2_O was further explored by Fourier transform infrared spectroscopy (FTIR). As displayed in Figure 2h, the vibration stretching peak of O─H blue‐shifted and the vibration bending peak of O─H red‐shifted after the addition of Met and Leu, indicating weakened hydrogen bonds.^[^ ^35^ ^]^ Met‐ZSO exhibited more obvious shift than Leu‐ZSO, which implies that Met had a strong interaction with H_2_O and decreased the hydrogen bond number of H_2_O, thus reducing free‐water activity in electrolytes and inhibiting possible HER.^[^ ^25^ ^]^ Furthermore, the strongest interaction between Met and H_2_O also endowed Met‐ZSO with the lowest freezing point among all the electrolytess (Figure S9, Supporting Information), thus leading to good cycling performance within Met‐ZSO at −8 °C.

**Figure 3:**
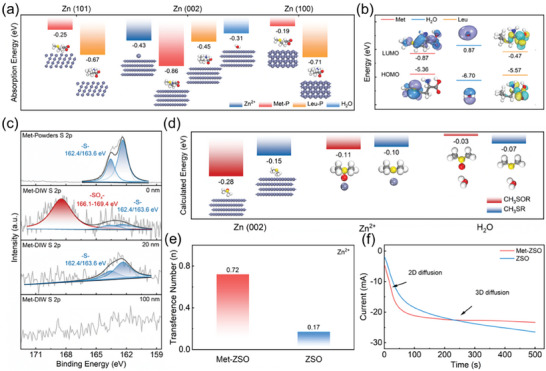
a) Absorption energy comparison of Met, Leu, H_2_O, and Zn^2+^ on different Zn crystal planes. b) HOMO‐LUMO energy levels of Met, Leu, and H_2_O. c) High‐resolution S2p XPS spectra of Met powders and Zn anode cycled in Met‐DIW by Ar^+^ sputtering for different depths. d) Calculation energy of CH_3_─S─R or CH_3_─SO─R with the Zn (002) crystal plane, Zn^2+^, or H_2_O. e) Zn^2+^ transference numbers of Zn anodes in Met‐ZSO and ZSO. f) CA curves in Met‐ZSO and ZSO.

DFT calculations were further employed to study the roles of additives on the Zn anode. As shown in **Figures** **3**a and S10 (Supporting Information), Met preferentially adsorbs on Zn (002) plane whether for parallel (P) absorption or vertical (V) absorption, while Leu tends to adsorb on Zn (100) and (101) planes. In addition, Met has a higher adsorption energy with Zn (002) plane (−0.86 eV) than with H_2_O (−0.31 eV) and with Zn^2+^ (−0.43 eV), indicating that Met would prefer to adsorb on Zn (002) plane before H_2_O and Zn^2+^ (Figure S11, Supporting Information). This result implies that although the entry of SO_4_^2−^ ions into the solvent sheath of Zn^2+^ seems to exacerbate corrosion in Met‐ZSO, Met prefers to form a protective layer and prevent continuous Zn corrosion on the Zn anode during cycling.^[^ ^21^ ^]^ In order to verify the inhibition effects of Zn corrosion, Zn foils were soaked in three electrolytess for 7 days and characterized by X‐ray diffraction (XRD, Figure S12, Supporting Information), which confirms that Met‐ZSO had the weakest characteristic peak of corrosion product (Zn_4_SO_4_OH_6_·3H_2_O). Notably, Leu‐ZSO led to the most corrosion products because of the relatively poorer adsorption energy between Leu and Zn foil as well as the largest number of SO_4_^2−^ coordinated with Zn^2+^, which is consistent with the MD and DFT simulation results.

Although it is proved that Met could spontaneously adsorb on Zn foil and form a protective layer to prevent Zn corrosion in electrolytess, the actual roles of Met played during charge‐discharge process should be further revealed due to the special electrochemical environment of Zn anode with electron injection and outflow. Therefore, the chemical stability of Met, Leu, and H_2_O were determined by DFT calculations (Figure 3b). It is shown that Met owns the highest HOMO energy level and the lowest LUMO energy level. This indicates that Met is most easily oxidized and reduced, thus contributing to the formation of SEI on the Zn anode. The chemical composition of the SEI on the Zn anode cycled in Met‐containing deionized water (Met‐DIW) was analyzed by X‐ray photoelectron spectroscopy (XPS) via sputtering with Ar^+^. Figure 3c shows that the C─S─C functional group of Met was oxidized into sulfoxide or sulfone functional group (C─SO_x_─C) on the SEI surface, which is also verified by the obvious characteristic peak of S═O located at 1020 cm^−1^ in the FTIR spectrum of cycled Zn anode in Met‐DIW (Figure S13, Supporting Information).^[^ ^36^ ^]^ To eliminate the possible oxidation by O_2_ in water or atmosphere, Met powders were dissolved in deionized water and placed in air for 24 h, followed by air drying at 80 °C. No C─SO_x_─C was detected in the treated Met powders by XPS, proving the electrochemical oxidation of Met during cycling (Figure S14, Supporting Information). Through Ar^+^ sputtering for different depth, it is shown that S composition gradually changed from C─SO_x_─C to C─S─C and finally disappeared after sputtering for 100 nm. Interestingly, DFT calculations (Figure 3d) show that C─SO_X_─C adsorbed on the Zn anode more easily than C─S─C. This suggests that the SEI should be formed by prior Met adsorption on Zn anode and subsequent electrochemical oxidation away from Zn anode. Moreover, C─SO_X_─C and C─S─C both have higher adsorption energy with Zn^2+^ than H_2_O, especially for C─SO_X_─C (−0.11 eV vs −0.03 eV), demonstrating the zincophilic and non‐hydrophilic properties of the unique SEI within Met‐ZSO. Furthermore, the SEI in Met‐ZSO possessed a higher transference number for Zn^2+^ than that in ZSO (0.72 vs 0.17, Figures 3e and S16, Supporting Information), which could be attributed to the accelerated Zn^2+^ desolvation in Met‐ZSO and boosted Zn^2+^ transport on Zn anode. The calculated activation energies (E_a_) are 33.2 and 40.2 kJ mol^−1^ on the Zn anodes in Met‐ZSO and ZSO, respectively, showing easier desolvation process and better reaction kinetics in Met‐ZSO than ZSO (Figure S17, Supporting Information).^[^ ^37^, ^38^ ^]^ Chronoamperometry (CA) was carried on to further study the Zn^2+^ diffusion processes in Met‐ZSO and ZSO (Figure 3f). When an overpotential of −150 mV was applied, the current density remained increasing over 500 s in ZSO, suggesting a long and rampant 2D diffusion process. In Met‐ZSO, the initial Zn nucleation and 2D diffusion processes occurred within 100 s, followed by a stable and constant 3D diffusion, revealing that the zincphilic gradient SEI could regulate the Zn^2+^ diffusion and deposition ways.^[^ ^22^ ^]^

**Figure 4:**
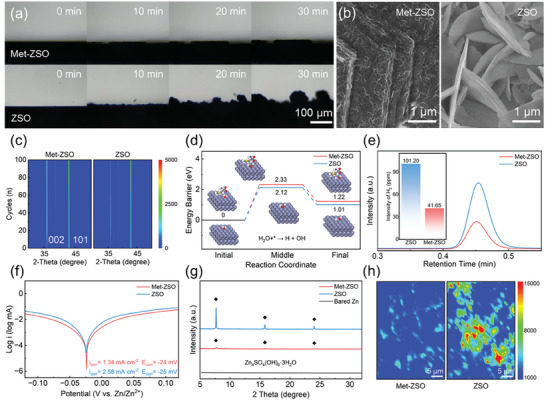
a) In situ optical images of the Zn plating processes in Met‐ZSO and ZSO under 5 mA cm^−2^. b) SEM images of Zn anodes after 20 cycles in Met‐ZSO (left) and ZSO (right). c) Ex situ XRD patterns of Zn anodes after different cycles in Met‐ZSO and ZSO. d) The dissociation energy barriers of H_2_O with and without Met additive. e) In‐situ EC‐GC profiles during Zn plating in Met‐ZSO and ZSO (inset: corresponding content of H_2_). f) Tafel plots of the Zn//Ti cells in Met‐ZSO and ZSO. g) XRD patterns and h) 2D Raman mapping images of Zn anodes after long cycling.

The Zn^2+^ deposition behavior was further observed by in situ optical microscopy under a constant current density of 5 mA cm^−2^ (**Figure** **4**a). It is shown that Met‐ZSO obviously inhibited the formation of Zn dendrite. The scanning electron microscope (SEM) images of the Zn anodes after 20 cycles (Figure 4b) show that Zn deposits exhibited obvious Zn (002) texture without dendrite in Met‐ZSO while numerous randomly oriented Zn nanoflakes were observed in ZSO. Besides, the Met‐derived SEI layer with low thickness could also observed on the anode surface. Ex‐situ XRD of Zn deposits on bared Zn further proved that the Met additives could ensure continuous plating/stripping in direction of Zn (002) plane and free growth of Zn (101) plane (Figure 4c). In addition to realize dendrite free, Met could also suppress HER during deposition. DFT calculations show that H_2_O dissociation has a higher energy barrier in Met‐ZSO than ZSO (Figure 4d). The linear sweep voltammetry (LSV, Figure S18, Supporting Information) displays decreased initial potential for HER after adding Met. In situ electrochemical gas chromatography (EC‐GC) was used to quantitatively monitor the H_2_ content during Zn plating at 5 mA cm^−2^. As shown in Figure 4e, the H_2_ release signal was markedly suppressed in Met‐ZSO with low H_2_ content. The good inhibition effect of HER contributed to long cycling performance, especially at room and high temperatures. Moreover, the Zn anode also possessed a lower corrosion current density (from 2.58 to 1.34 mA cm^−2^) in Met‐ZSO than ZSO (Figure 4f; Figure S19, Supporting Information). After long cycling in Zn//Zn cells, much fewer corrosion products on the surface of Zn anode could be detected after the addition of Met (Figure 4g; Figure S20, Supporting Information), verifying the good inhibition effect on Zn corrosion. It should be noted that HER and corrosion reactions were also easily occurred on the Zn anodes in Leu‐ZSO (Figures S18 and S19, Supporting Information). However, Leu could form SEI as protective layer to inhibition continuous corrosion during cycling, thus leading to fewer corrosion productions in Leu‐ZSO than ZSO (Figure S20, Supporting Information).^[^ ^35^ ^]^ The 2D Raman mapping images (dominated peak at 964 cm^−1^, corresponding to corrosion products) further indicate that numerous by‐products were unevenly dispersed on the cycled Zn anode in ZSO, while only a few by‐products were found on the cycled Zn anode in Met‐ZSO (Figure 4h; Figure S21, Supporting Information).^[^ ^12^ ^]^

**Figure 5:**
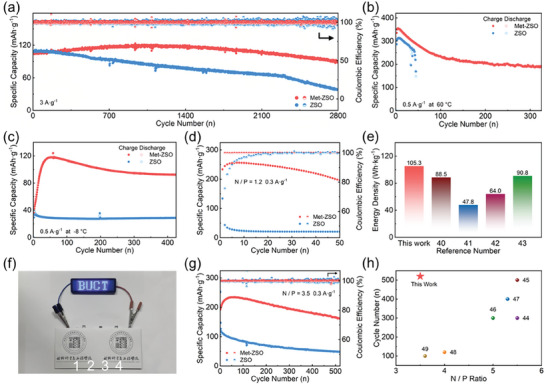
a) Cycling performance of the Zn//NVO full cells in Met‐ZSO and ZSO under 3 A g^−1^ using excess Zn. Cycling performance of the Zn//NVO full cells in Met‐ZSO and ZSO under 0.5 A g^−1^ using excess Zn at b) 60 °C and c) −8 °C. d) Cycling performance of the Zn//NVO full cells in Met‐ZSO and ZSO under 0.3 A g^−1^ with limited Zn (N/P = 1.2). e) Comparison of energy density of the full cell using Met‐ZSO with recent literature studies. f) Optical image of LEDs powered by four Zn//NVO pouch cells using Met‐ZSO. g) Cycling performance of the Zn//NVO full cells in Met‐ZSO and ZSO under 0.3 A g^−1^ with limited Zn (N/P = 3.5). h) Comparison of cycling number and N/P ratio for the full cell using Met‐ZSO with recent literature studies.

NH_4_V_4_O_10_ (NVO) was prepared by a hydrothermal method (Figures S22 and S23, Supporting Information),^[^ ^39^ ^]^ and used as cathode to assemble full cells with ZSO and Met‐ZSO. When excess Zn anode was employed, the Zn//NVO full cell using Met‐ZSO delivered a high discharge capacity of 294.8 mA h g^−1^ under 0.3 A g^−1^, which maintained 104.4 mA h g^−1^ at 5 A g^−1^, exhibiting a better rate performance than the cell using ZSO (Figures S24 and S25, Supporting Information). Moreover, Met‐ZSO endowed the Zn//NVO full cells with excellent cycling performance in wide temperature range. At room temperature, the full cell could stably work for over 2800 cycles under 3 A g^−1^ (**Figure** **5**a). Even at −8 and 60 °C the Zn//NVO full cell using Met‐ZSO still showed good cycling performance over 300 and 400 cycles, and provided maximum specific capacities of 350 and 120 mA h g^−1^, respectively (Figure 5b,c). To further estimate practical performance, Zn//NVO full cells with low N/P ratio were also assembled using Met‐ZSO. The full cell using Met‐ZSO with N/P ratio of 1.2 could delivered a high energy density of 105.30 W  h kg^−1^ (Figure S26, Supporting Information) and exhibited much higher capacity and better cycling performance than the cell using ZSO (Figure 5d). The energy density is higher than those in literature works (Figure 5e).^[^ ^40^, ^41^, ^42^, ^43^ ^]^ Therefore, pouch cells using Met‐ZSO could easily power 140 light‐emitting diodes (LEDs) (Figure 5f). When the N/P ratio increased to 3.5, the full cell using Met‐ZSO could realize a good cycling performance over 500 cycles (Figure 5g), which is superior to the recent AZMBs with limited N/P ratio (Figure 5h),^[^ ^44^, ^45^, ^46^, ^47^, ^48^, ^49^ ^]^ demonstrating great commercial potential of Met‐ZSO in AZMBs.

## 3 Conclusion

In summary, we used Met as an efficient electrolytes additive for AZMBs. The different roles of Met additive played in modifying Zn anode were detailly revealed from electrolytes to electrodes. The Met additive could synergistically suppress HER/corrosion reactions and guide dendrite‐free Zn deposition. Therefore, the Met‐containing electrolytes endowed half cells and full cells with good performance even under extreme test conditions, showing a good prospect of Met‐ZSO for practical use in AZMBs. This work not only gives a new insight into the modification mechanism of sulfur‐containing zwitter‐molecule for Zn anode, but also provides a new electrolytes additive for the development of practical AZMBs.

## Conflict of Interest

The authors declare no conflict of interest.

## Supporting information

Supporting Information

## Data Availability

The data that support the findings of this study are available from the corresponding author upon reasonable request.

## References

[1] J. Zheng , L. A. Archer , Sci. Adv. 2021, 7, eabe0219. 33523975 10.1126/sciadv.abe0219 PMC7787491

[2] S. Wu , Z. Hu , P. He , L. Ren , J. Huang , J. Luo , eScience 2023, 3, 100120.

[3] S. Liu , R. Zhang , J. Mao , Y. Zhao , Q. Cai , Z. Guo , Sci. Adv. 2022, 8, eabn5097. 35319992 10.1126/sciadv.abn5097 PMC8942368

[4] H. L. Pan , Y. Y. Shao , P. F. Yan , Y. W. Cheng , K. S. Han , Z. M. Nie , C. M. Wang , J. H. Yang , X. L. Li , P. Bhattacharya , K. T. Mueller , J. Liu , Nat. Energy 2016, 1, 16039.

[5] X. Yu , Z. Li , X. Wu , H. Zhang , Q. Zhao , H. Liang , H. Wang , D. Chao , F. Wang , Y. Qiao , H. Zhou , S.‐G. Sun , Joule 2023, 7, 1145.

[6] T. C. Li , Y. Lim , X. L. Li , S. Luo , C. Lin , D. Fang , S. Xia , Y. Wang , H. Y. Yang , Adv. Energy Mater. 2022, 12, 2103231.

[7] Z. Cai , J. Wang , Y. Sun , eScience 2023, 3, 100093.

[8] Y. Zuo , K. Wang , P. Pei , M. Wei , X. Liu , Y. Xiao , P. Zhang , Mater. Today Energy 2021, 20, 100692.

[9] T. Wang , C. Li , X. Xie , B. Lu , Z. He , S. Liang , J. Zhou , ACS Nano 2020, 14, 16321. 33314908 10.1021/acsnano.0c07041

[10] Z. Li , A. W. Robertson , Battery Energy 2022, 2, 20220029.

[11] S. B. Wang , Q. Ran , R. Q. Yao , H. Shi , Z. Wen , M. Zhao , X. Y. Lang , Q. Jiang , Nat. Commun. 2020, 11, 1634. 32242024 10.1038/s41467-020-15478-4 PMC7118111

[12] Z. Zhao , R. Wang , C. Peng , W. Chen , T. Wu , B. Hu , W. Weng , Y. Yao , J. Zeng , Z. Chen , P. Liu , Y. Liu , G. Li , J. Guo , H. Lu , Z. Guo , Nat. Commun. 2021, 12, 6606. 34785684 10.1038/s41467-021-26947-9 PMC8595410

[13] H. Yu , Y. Zeng , N. W. Li , D. Luan , L. Yu , X. W. Lou , Sci. Adv. 2022, 8, eabm5766. 35275713 10.1126/sciadv.abm5766 PMC8916735

[14] Y. S. Meng , V. Srinivasan , K. Xu , Science 2022, 378, eabq3750. 36480630 10.1126/science.abq3750

[15] P. Sun , L. Ma , W. Zhou , M. Qiu , Z. Wang , D. Chao , W. Mai , Angew. Chem., Int. Ed. 2021, 60, 18247. 10.1002/anie.202105756 34036748

[16] Q. Meng , R. Zhao , P. Cao , Q. Bai , J. Tang , G. Liu , X. Zhou , J. Yang , Chem. Eng. J. 2022, 447, 137471.

[17] B. Wang , R. Zheng , W. Yang , X. Han , C. Hou , Q. Zhang , Y. Li , K. Li , H. Wang , Adv. Funct. Mater. 2022, 32, 2112693.

[18] Z. Cao , X. Zhu , S. Gao , D. Xu , Z. Wang , Z. Ye , L. Wang , B. Chen , L. Li , M. Ye , J. Shen , Small 2022, 18, e2103345. 34862723 10.1002/smll.202103345

[19] J. Hao , L. Yuan , C. Ye , D. Chao , K. Davey , Z. Guo , S. Z. Qiao , Angew. Chem., Int. Ed. 2021, 60, 7366. 10.1002/anie.202016531 33440043

[20] D. Feng , F. Cao , L. Hou , T. Li , Y. Jiao , P. Wu , Small 2021, 17, e2103195. 34528386 10.1002/smll.202103195

[21] C. Meng , W. He , L. Jiang , Y. Huang , J. Zhang , H. Liu , J. J. Wang , Adv. Funct. Mater. 2022, 32, 2207732.

[22] Y. Lin , Z. Mai , H. Liang , Y. Li , G. Yang , C. Wang , Energy Environ. Sci. 2023, 16, 687.

[23] R. Sun , D. Han , C. Cui , Z. Han , X. Guo , B. Zhang , Y. Guo , Y. Liu , Z. Weng , Q. H. Yang , Angew. Chem., Int. Ed. 2023, 62, e202303557. 10.1002/anie.202303557 37191972

[24] Z. Hu , F. Zhang , Y. Zhao , H. Wang , Y. Huang , F. Wu , R. Chen , L. Li , Adv. Mater. 2022, 34, 2203104. 10.1002/adma.202203104 35765154

[25] J. Wan , R. Wang , Z. Liu , L. Zhang , F. Liang , T. Zhou , S. Zhang , L. Zhang , Q. Lu , C. Zhang , Z. Guo , ACS Nano 2023, 17, 1610. 10.1021/acsnano.2c11357 36594407

[26] R. Zhao , H. Wang , H. Du , Y. Yang , Z. Gao , L. Qie , Y. Huang , Nat. Commun. 2022, 13, 3252. 35668132 10.1038/s41467-022-30939-8 PMC9170708

[27] H. M. Yu , D. P. Chen , X. Y. Ni , P. Qing , C. S. Yan , W. F. Wei , J. M. Ma , X. B. Ji , Y. J. Chen , L. B. Chen , Energy Environ. Sci. 2023, 16, 2684.

[28] H. Wang , W. Ye , B. Yin , K. Wang , M. S. Riaz , B. B. Xie , Y. Zhong , Y. Hu , Angew. Chem., Int. Ed. 2023, 62, e202218872. 10.1002/anie.202218872 36647214

[29] J. Yang , Y. Zhang , Z. Li , X. Xu , X. Su , J. Lai , Y. Liu , K. Ding , L. Chen , Y. P. Cai , Q. Zheng , Adv. Funct. Mater. 2022, 32, 2209642.

[30] C. Huang , X. Zhao , S. Liu , Y. Hao , Q. Tang , A. Hu , Z. Liu , X. Chen , Adv. Mater. 2021, 33, 2100445. 10.1002/adma.202100445 34338350

[31] Y. Zhong , Z. X. Cheng , H. W. Zhang , J. B. Li , D. D. Liu , Y. Q. Liao , J. T. Meng , Y. Shen , Y. H. Huang , Nano Energy 2022, 98, 107220.

[32] M. Yan , N. Dong , X. Zhao , Y. Sun , H. Pan , ACS Energy Lett. 2021, 6, 3236.

[33] S. Zhao , Y. Zuo , T. Liu , S. Zhai , Y. Dai , Z. Guo , Y. Wang , Q. He , L. Xia , C. Zhi , J. Bae , K. Wang , M. Ni , Adv. Energy Mater. 2021, 11, 2101749.

[34] J. Xu , W. Lv , W. Yang , Y. Jin , Q. Jin , B. Sun , Z. Zhang , T. Wang , L. Zheng , X. Shi , B. Sun , G. Wang , ACS Nano 2022, 16, 11392. 35848633 10.1021/acsnano.2c05285

[35] Y. Liu , Y. An , L. Wu , J. Sun , F. Xiong , H. Tang , S. Chen , Y. Guo , L. Zhang , Q. An , L. Mai , ACS Nano 2023, 17, 552. 36524731 10.1021/acsnano.2c09317

[36] P. P. Kannan , N. K. Karthick , A. Mahendraprabu , R. Shanmugam , A. Elangovan , G. Arivazhagan , J. Mol. Struct. 2017, 1139, 196. 10.1016/j.saa.2017.01.068 28199926

[37] Y. Zhou , G. Li , S. Feng , H. Qin , Q. Wang , F. Shen , P. Liu , Y. Huang , H. He , Adv. Sci. 2023, 10, e2205874. 10.1002/advs.202205874 PMC9951317 36574480

[38] C. Tian , J. Wang , R. Sun , T. Ali , H. Wang , B. B. Xie , Y. Zhong , Y. Hu , Angew. Chem., Int. Ed. 2023, 62, e202310970. 10.1002/anie.202310970 37644643

[39] Y. Fang , X. Xie , B. Zhang , Y. Chai , B. Lu , M. Liu , J. Zhou , S. Liang , Adv. Funct. Mater. 2021, 32, 2109671.

[40] H. Yan , S. Li , H. Xu , H. Chen , S. Yang , B. Li , Adv. Energy Mater. 2022, 12, 2201599.

[41] M. Zhu , Q. Ran , H. Huang , Y. Xie , M. Zhong , G. Lu , F. Q. Bai , X. Y. Lang , X. Jia , D. Chao , Nanomicro. Lett. 2022, 14, 219. 36355311 10.1007/s40820-022-00969-4 PMC9649586

[42] J. Luo , L. Xu , Y. Zhou , T. Yan , Y. Shao , D. Yang , L. Zhang , Z. Xia , T. Wang , L. Zhang , T. Cheng , Y. Shao , Angew. Chem., Int. Ed. 2023, 62, e202302302. 10.1002/anie.202302302 36959698

[43] G. Ma , S. Di , Y. Wang , W. Yuan , X. Ji , K. Qiu , M. Liu , X. Nie , N. Zhang , Energy Storage Mater. 2023, 54, 276.

[44] Y. Zou , Y. Su , C. Qiao , W. Li , Z. Xue , X. Yang , M. Lu , W. Guo , J. Sun , Adv. Energy Mater. 2023, 13, 2300932.

[45] C. Huang , X. Zhao , Y. Hao , Y. Yang , Y. Qian , G. Chang , Y. Zhang , Q. Tang , A. Hu , X. Chen , Energy Environ. Sci. 2023, 16, 1721.

[46] Z. Hou , Y. Gao , H. Tan , B. Zhang , Nat. Commun. 2021, 12, 3083. 34035276 10.1038/s41467-021-23352-0 PMC8149847

[47] C. Huang , F. Huang , X. Zhao , Y. Hao , Y. Yang , Y. Qian , G. Chang , Y. Zhang , Q. Tang , A. Hu , X. Chen , Adv. Funct. Mater. 2022, 33, 2210197.

[48] P. X. Xiong , C. Y. Lin , Y. Wei , J. H. Kim , G. Jang , K. R. Dai , L. X. Zeng , S. P. Huang , S. J. Kwon , S. Y. Lee , H. S. Park , ACS Energy Lett. 2023, 8, 2718.

[49] X. Bai , Y. Nan , K. Yang , B. Deng , J. Shao , W. Hu , X. Pu , Adv. Funct. Mater. 2023, 33, 2307595.

