# Supplementary material for: Insight into Sulfur‐Containing Zwitter‐Molecule Boosting Zn Anode: from Electrolytes to Electrodes
Source: Adv Sci (Weinh). 2024 Feb 23;11(17):2400094. doi: 10.1002/advs.202400094 (PMC11077684; doi:10.1002/advs.202400094)
Supplement: Supplementary file 1 — Supporting Information [file ADVS-11-2400094-s001.pdf]

ADVANCED SCIENCE
Open Access

Supporting Information

for *Adv. Sci.*, DOI 10.1002/advs.202400094

Insight into Sulfur-Containing Zwitter-Molecule Boosting Zn Anode: from Electrolytes to Electrodes

Weihao Song, Jiaxing Liu, Shengpu Rao, Ming Zhao, Yanqun Lv, Shunshun Zhao, Qing Ma, Bing Wu, Chengjin Zheng, Shimou Chen, Zhilin Li, Jin Niu* and Feng Wang**

Supporting Information

Insight into Sulfur-Containing Zwitter-Molecule Boosting Zn Anode: from Electrolyte to Electrode

Weihao Song, [a, b, #] Jiaxing Liu, [a, b, #] Shengpu Rao,[a, b] Ming Zhao, [a] Yanqun Lv, [a] Shunshun Zhao, [a] Qing Ma, [a, b] Bing Wu, [a, b] Chengjin Zheng, [a, b] Shimou Chen, [a] Zhilin Li, [a, b, *] Jin Niu, [a, b, *] and Feng Wang [a, b, *]

a State Key Laboratory of Chemical Resource Engineering, Laboratory of Electrochemical Process and Technology for materials, Beijing University of Chemical Technology, Beijing, 100029, P. R. China

b Beijing Advanced Innovation Center for Soft Matter Science and Engineering, Beijing University of Chemical Technology, Beijing, 100029, P. R. China

[#] These authors contributed equally to this work.

* Corresponding authors.

E-mail: lizl@buct.edu.cn; niujin@buct.edu.cn; wangf@buct.edu.cn

Material preparation

The ZSO (2 M) was prepared using $ZnSO_4 \cdot 7H_2O$ (Sinopharm Chemical Reagent Co., Ltd) and deionized water. Electrolytes with additives were prepared by adding 10 mM Met (Adamas) and Leu (J&K Scientific) powders into 2 M $ZnSO_4$ aqueous solutions. Na_2SO_4 was purchased from Aladdin. The diameter of the Zn plates is 14 mm with a thickness of 100 μm. Before use, the Zn plates were polished using sandpapers (2000 meshes), followed by water/ethanol washing for several times. The electrolyte

amount for the cell were 100 μL. As for the cathode materials, 10 mM NH_4VO_3 (Macklin) was dissolved in 80 °C deionized water. Subsequently, 15 mM $H_2C_2O_4{\cdot}2H_2O$ (Bidepharm) solid powders were added to the solution. The solution was transferred to a 100 mL hydrothermal reactor and kept in an oven at 140 °C for 48h. The products were collected and washed repeatedly with deionized water until neutral after the sample was cooled to room temperature naturally. The final product was dried at 80 °C to obtain the NVO.[1]

Material Characterization

The structural formulas were drawn by KingDraw. The morphology of materials and anodes were characterized by SEM (JEOL, JSM-6701F). XRD patterns were recorded on a SHIMADZU XRD diffra-7000 X-RAY diffractometer. Raman spectra were characterized using a LabRam HR800 spectrometer. FTIR spectra were measured by a PerkinElmer Spectrum 100 spectrometer. FTIR was performed on a Nicolet Nexus 670 spectrometer. Raman spectra were recorded using the inVia Reflex. XPS was performed using the Thermo Scientific K-Alpha spectrometer equipped with Al Kα radiation (1486.6 eV). All the XPS spectra were calibrated with the binding energy of sp^2 carbon in the C1s spectra as 284.80 eV. In-situ optical microscopy analyses were carried on an optical microscope (XJ-550). H_2 evolution was quantified by in-situ electrochemical gas chromatography (SHIMADZU GC-2030). DSC was assisted with MPMS in determining the freezing point of the electrolytes (Netzsch, DSC 200F3, U.S.A.) from 30 °C to -30 °C.

Electrochemical Measurements

The 2032-type coin cells were used for all the electrochemical tests in this work. All the cycling tests were conducted on a LAND (CT2001A, China) testing system. The glass fiber membrane (Whatman GF/D, GE Healthcare) was used as the separator. The thick electrodes of NVO were prepared by mixing 70 wt.% of active material, 20 wt.% of Kenjen black and 10 wt.% of polytetrafluoroethylene in ethanol to obtain a paste. Then, the film was pressed into a stainless steel mesh for electrochemical testing. The areal mass loading of NVO electrodes was about 3~4 mg cm^{-2}. The full cells of Zn//NVO were assembled using Zn foils as the anode and NVO as the cathode. All the full cells were cycled in the range of 0.4 to 1.4 V using the ZSO and Met-ZSO. The pouch cells of Zn//NVO with an area of 25 cm^2 (5 cm × 5 cm) were assembled. LSV, cyclic voltammetry (CV), Tafel plots, electrochemical impedance spectroscopy (EIS), and CA were measured on an electrochemical workstation (CHI 660E, China). More specifically, LSV, Tafel, and CV curves were measured at a scan rate of 5 mV s^{-1} in different half cells (Zn//Zn or Zn//Ti). CA curves were tested at a constant potential (-150 mV). The H_2 evolution was detected via in situ EC-GC. A glass bottle with two Zn-foils (1 cm × 1 cm) were applied for EC-GC measurement. The current and persistent time in Zn plating are fixed at 5 mA and 0.25 h, respectively.[2] The Zn^{2+} transference numbers were obtained according to the typical Evans method, which should be described as the following formula:

$$t_{Zn^{2+}} = \frac{I_s(\Delta V - I_0 R_0)}{I_0(\Delta V - I_s R_s)}$$

where I_0/I_s and R_0/R_s represent the initial/final current density (tested by CA) and charge transfer resistance (tested by EIS) before and after the polarization test, ΔV is

the constant polarization potential for the CA test.[3] The desolvation energy (E_a) was obtained according to the Arrhenius equation:

$$\frac{1}{R_{ct}} = Ae^{-\frac{E_a}{RT}}$$

where R_{ct} is the charge transfer resistance, A is the pre-exponential constant, T is the absolute temperature, and R is the standard gas constant. Measurement temperatures are 20, 30, 40, 50, and 60 °C.[4-6] Besides, the Zn//NVO full cells were cycled at a constant rate within the voltage range of 0.4 to 1.4 V. The energy density E (W h kg^{-1}) was calculated using the following equations:

$$E = \int IV\,dt$$

where I is the current density (A kg^{-1}), V is the voltage (V), and t is the discharge time (h). The weight is based on the total mass of the active material and Zn anode (mg).

DFT calculations

The absorption energy and binding energy were calculated using DFT implemented with the $DMol^3$ package in the Materials Studio 2017. The Perdew-Burke-Ernzerhof (PBE) exchange correlation functional within the generalized gradient approximation (GGA) was used to describe the exchange-correlation energy. The adsorption energy was calculated with the equation:

$$\Delta E = E_{a-b} - (E_a + E_b)$$

where ΔE is the adsorption energy, and E_{a-b} is the total energy of the relaxed a and b models at the equilibrium state. E_a and E_b are the self-consistent field (SCF) calculation energy values of geometry-optimized a and b models. Electron exchange correlation was constructed by PBE function with generalized gradient approximation

GGA in the Materials studio 2017.[7] In all calculations, a cutoff energy with the value of 400 eV was used for the plane wave basis, and the convergence criteria for the ionic relaxation and the electronic self-consistent calculation were set to 0.02 eV Å^{-1} and 10^{-5} eV. The Brillouin zone integration was performed with 3 × 3 × 1 k-points. It should be noted that two simple models (Figure 3d) were used to represent CH_3-S-R and CH_3-SO-R for calculations. For the dissociation process of H_2O, the dissociation energy was calculated by equation:

$$\Delta E = E_{FS} - E_{IS}$$

where E_{IS} and E_{FS} were the total energies of reactant and final state directly from DFT calculations.[8-9]

HOMO-LUMO and ESP analyses were calculated with DMol^3 package in the Materials Studio 2017.

MD calculations

MD simulations were conducted on the electrolytes using the Materials Studio 2017. The box size of the samples was 27 × 27 × 27 Å^3, which included 550 water molecules, 20 Zn^{2+} ions, and 20 SO_4^{2-} ions. The additive molecules were according to the proportion of Met and Leu. The COMPASS II force fields were selected for assigning charges. Moreover, a potential cutoff radius of 12.5 Å was applied within the calculation of the nonbonded interaction. First, the steepest descent method and energy minimization were performed to obtain a stable structure in the Forcite module. Subsequently, all mixture systems were equilibrated at NVT for 50 ps at 298 K, followed by NPT for 100 ps with a 1 fs time step. And 50-100ps was used to analyze

RDFs. The temperature and pressure coupling were performed in Nose-Hoover and Berendsen methods, respectively.[10-15] The RDFs give the probability of molecules occurring at the distance (r),[16] which is calculated by:

$$g(r) = \frac{1}{4\pi r^2 \rho N} \sum_{i=1}^{N} \sum_{j=1, j \neq i}^{N} \delta\left(r - |r_{ij}|\right)$$

where ρ is the density of particles, N is the total number of all particles in the simulation box and $|r_{ij}|$ is the interatomic distance between particle i and particle j Based on the $g(r)$, the coordination number CN can be calculated by:

$$CN = \int_{r_1}^{r_2} 4\pi r^2 \, g(r) \rho \, dr$$

where r_1 and r_2 are the r range for the calculation of CN in the first solvation shell. According to the $g(r)$ in Figure 2d-f, the r_1 and r_2 for CN of the first solvation shell are set to be 0 and 3 Å, respectively.[17]

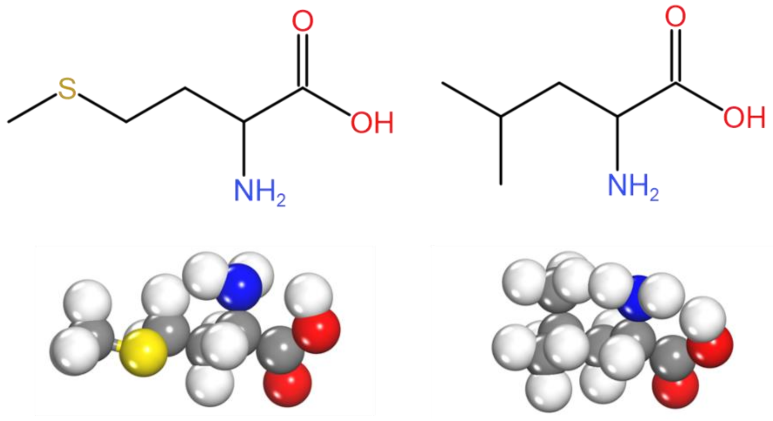

Figure S1. Structural formulas and spatial structures of Met (left) and Leu (right).

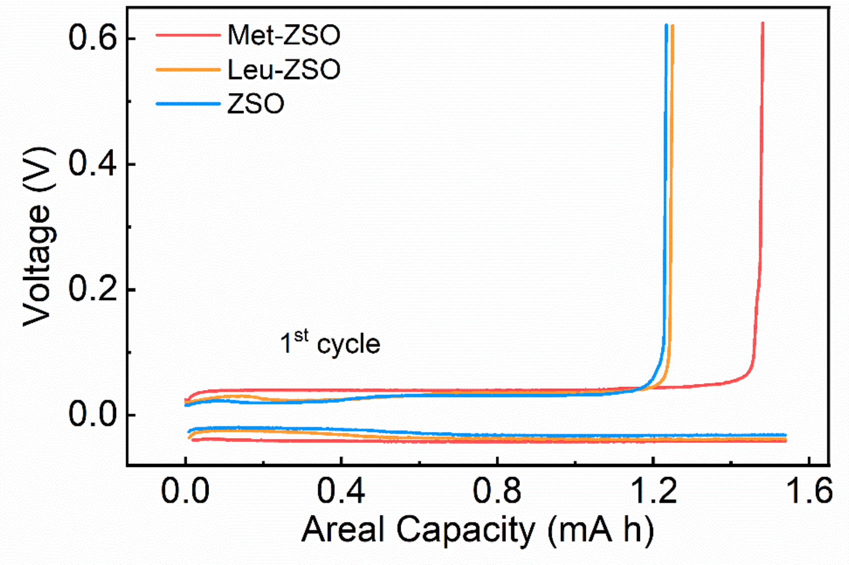

Figure S2. Initial Coulombic efficiency of Zn//Cu cells in different electrolytes under 2 mA cm^{-2} with 1 mA h cm^{-2}.

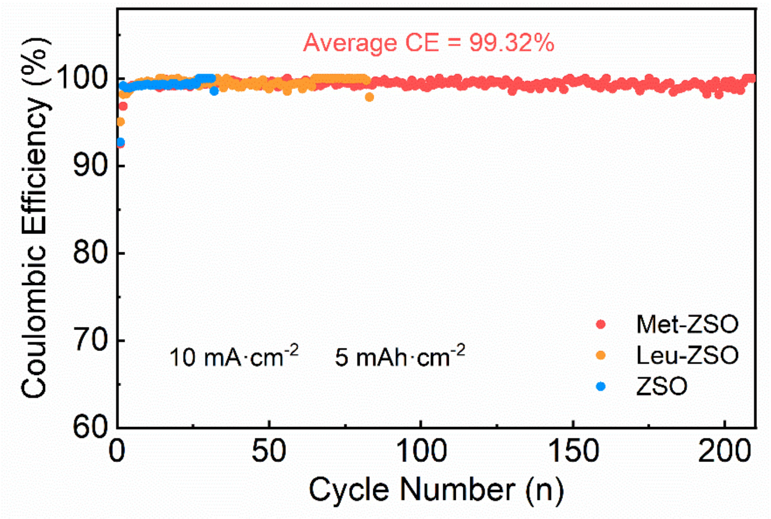

Figure S3. Coulombic efficiency of Zn//Cu cells in different electrolytes under 10 mA cm^{-2} with 5 mA h cm^{-2}.

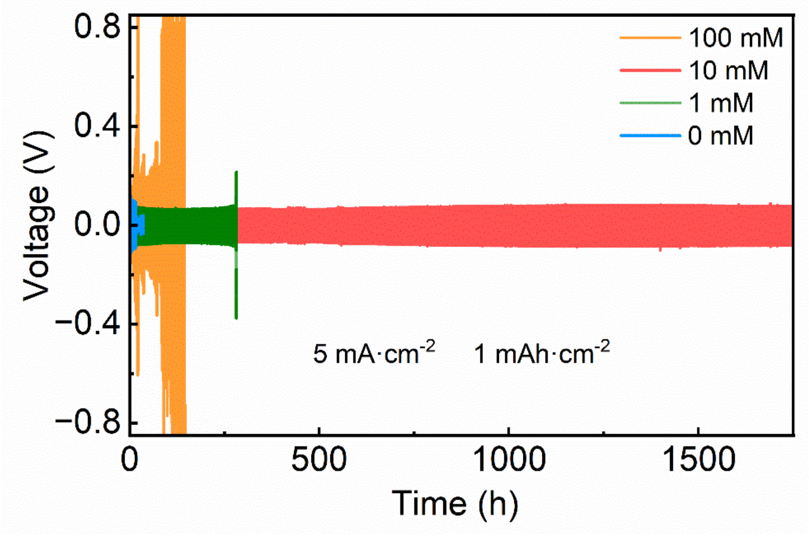

Figure S4. Cycling performance of Zn//Zn cells in Met-ZSO with different addition amounts.

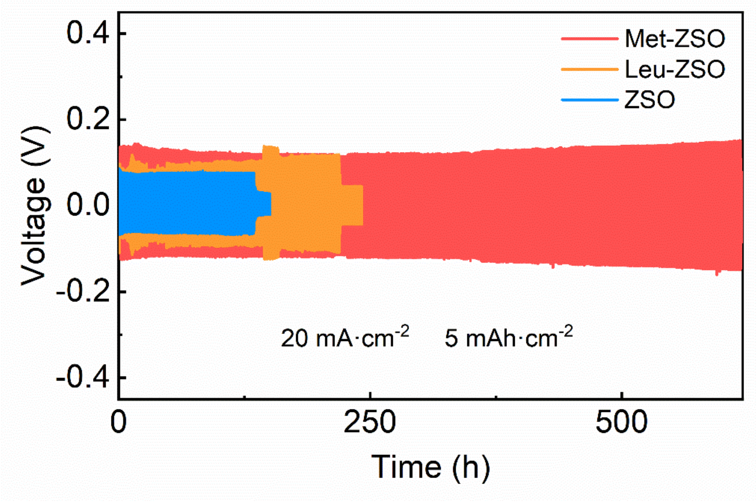

Figure S5. Cycling performance of Zn//Zn cells in different electrolytes under 20 mA cm^{-2} with 5 mA h cm^{-2}.

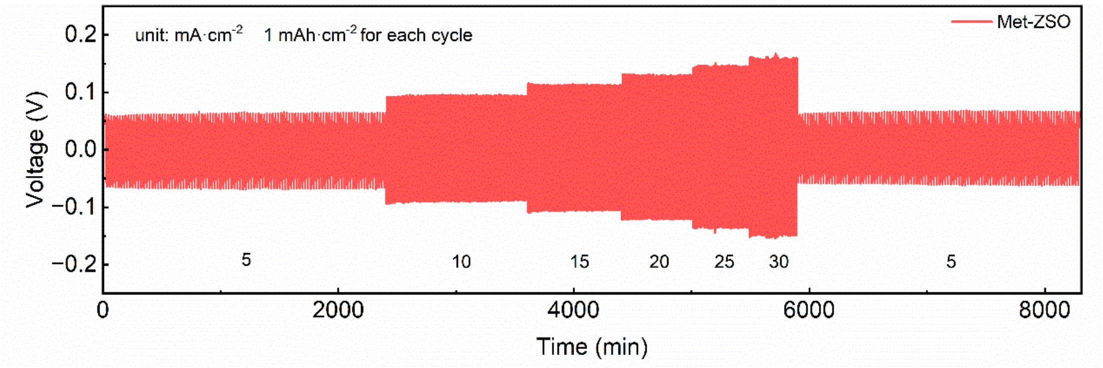

Figure S6. Rate performance of Zn//Zn cell in Met-ZSO.

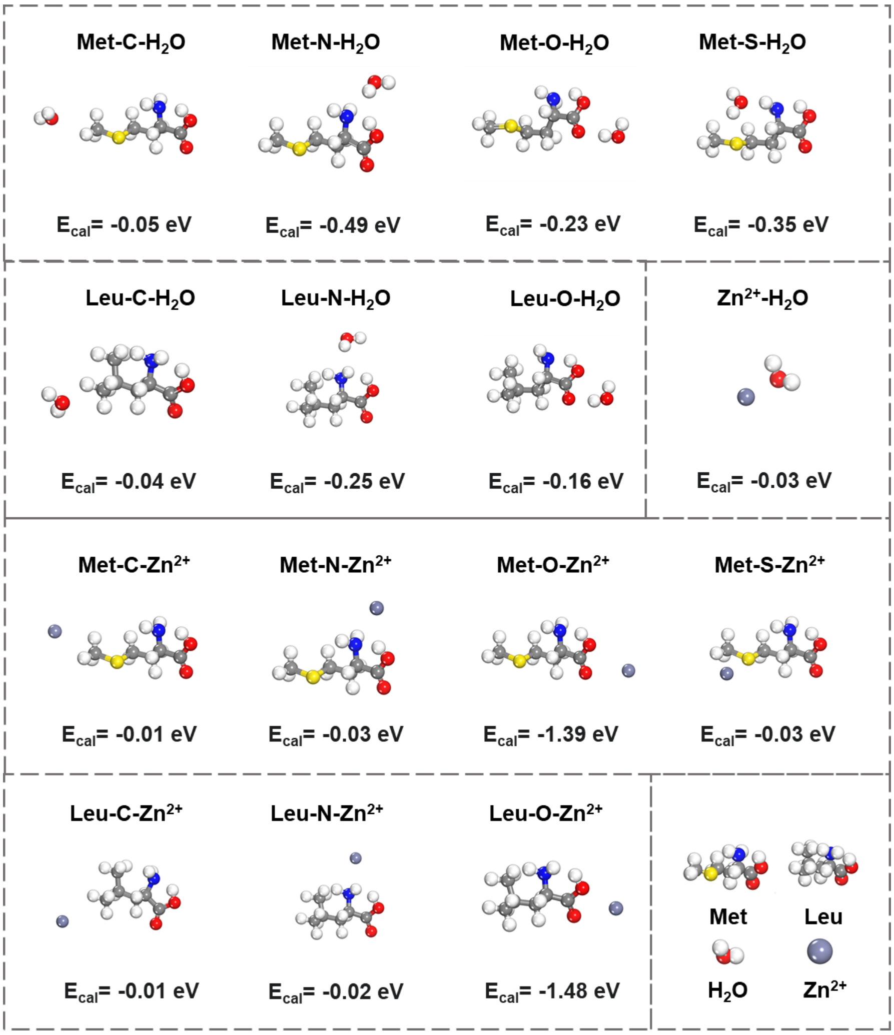

Figure S7. DFT calculations of binding energy of H_2O, Zn^{2+}, Leu, and Met.

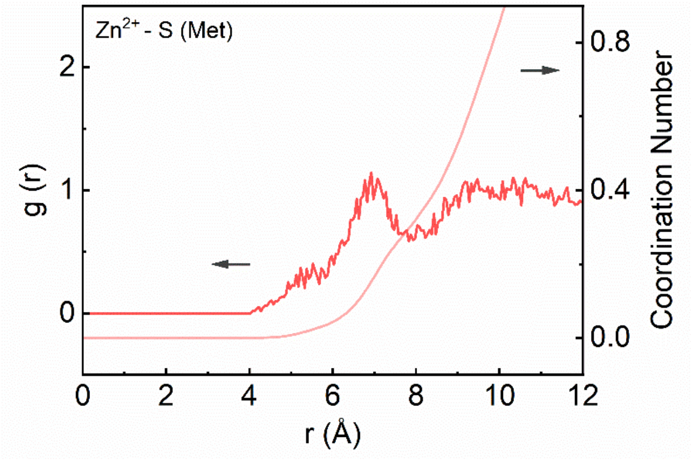

Figure S8. RDFs for Zn^{2+}-S (Met) collected from MD simulations in Met-ZSO.

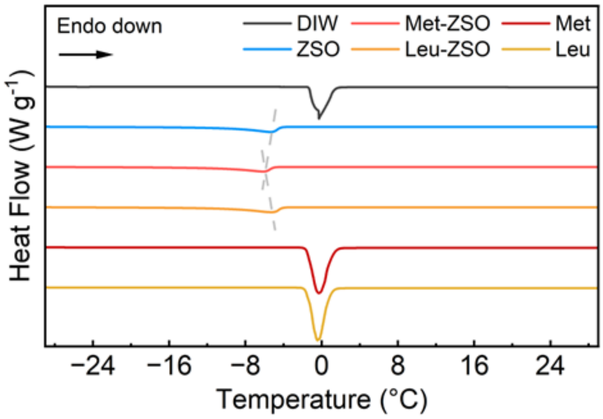

Figure S9. DSC curves of various liquid.

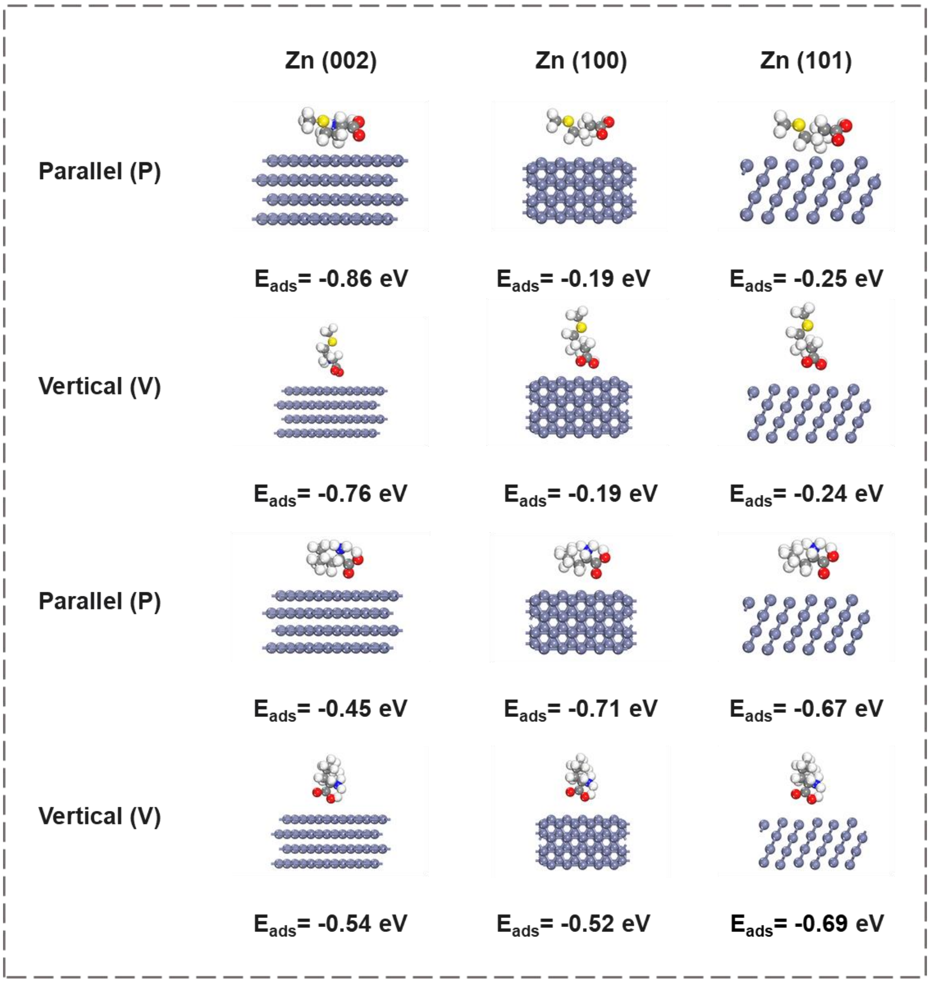

Figure S10. DFT calculations of adsorption energy on different Zn plane of Leu and Met.

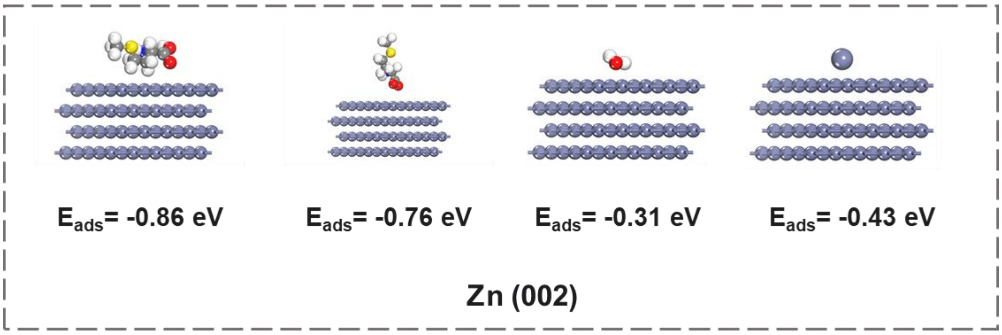

Figure S11. DFT calculations of adsorption energy on Zn (002) plane of Met, H_2O, and Zn^{2+}.

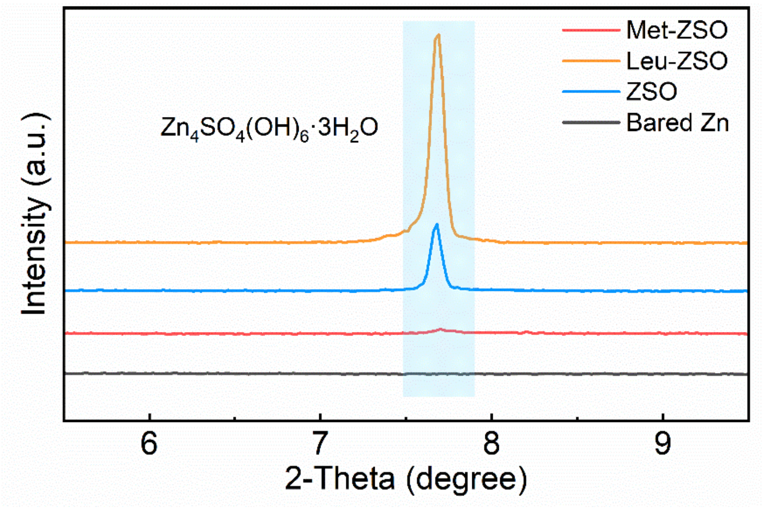

Figure S12. XRD patterns of Zn anode after soaking in three electrolytes for 7 days.

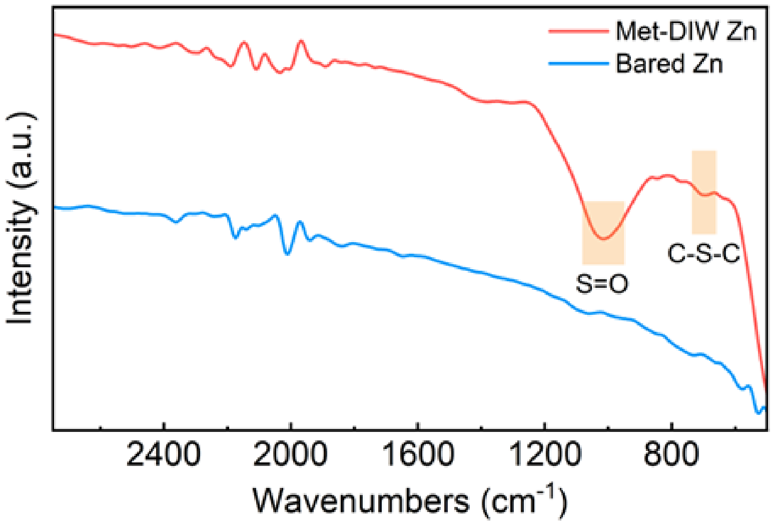

Figure S13. FTIR spectra of Zn anode cycled in Met-DIW electrolyte for 50 cycles.

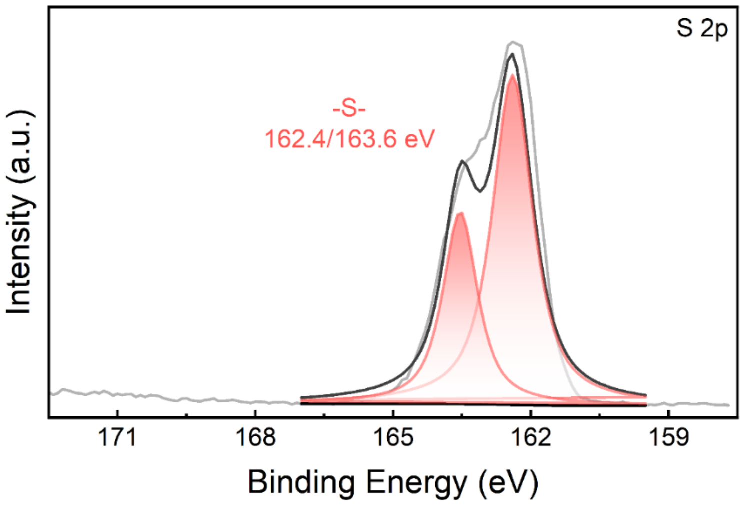

Figure S14. High-resolution S 2p XPS spectra of Met powders after dissolving in deionized water and drying overnight in outdoor oven at 80°C.

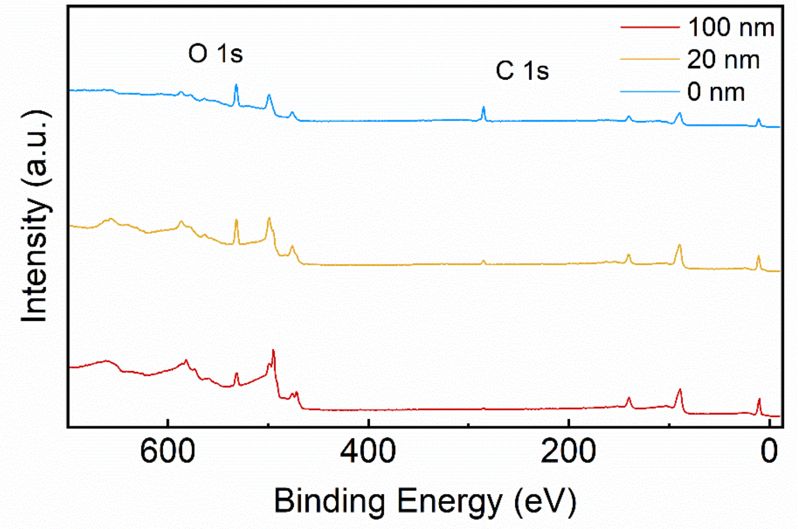

Figure S15. XPS spectra of Zn anode (cycled in Met-DIW electrolyte for 50 cycles) with different sputtering depths.

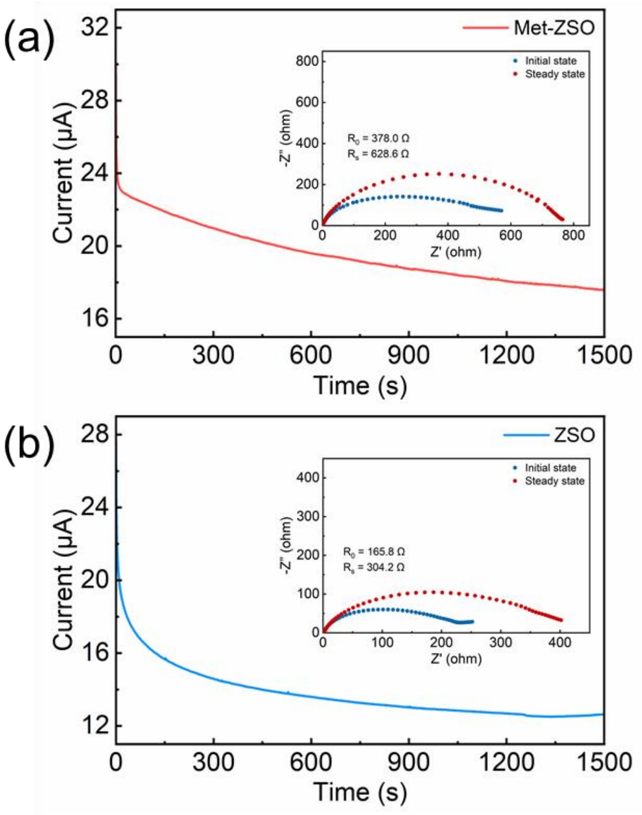

Figure S16. CA curves at an applied voltage of 10 mV under the room temperature, inset: EIS collected from Zn//Zn cells before and after polarization operations in (a) Met-ZSO and (b) ZSO.

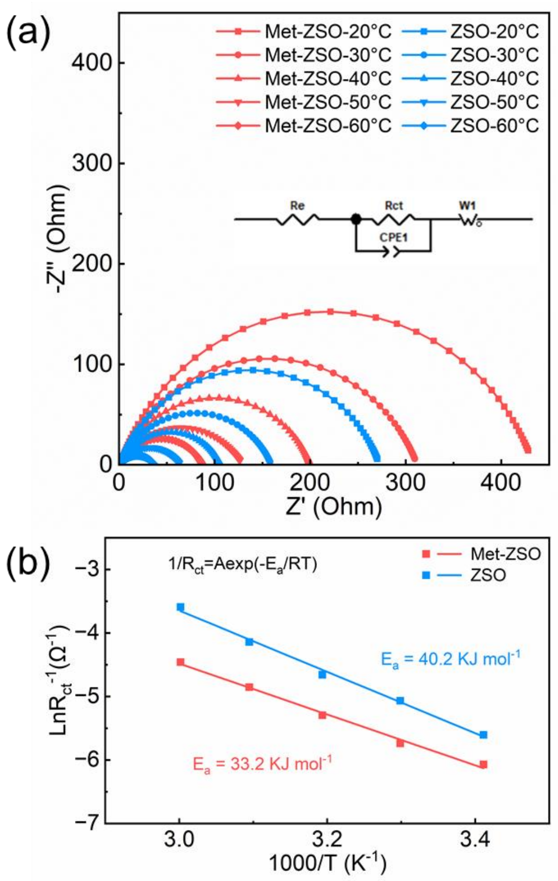

Figure S17. (a) Nyquist plots at different temperatures for the Zn//Zn cells in Met-ZSO and ZSO after cycling (inset: fitting circuit of the Zn//Zn cells). (b) Corresponding Arrhenius curves and comparison of activation energies.

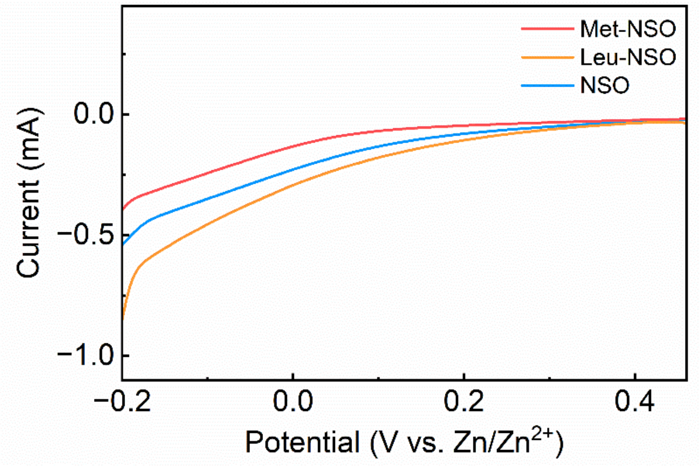

Figure S18. LSV curves of the Zn//Ti cells in the Na_2SO_4 electrolyte (NSO) with Met, Leu, and no additive.

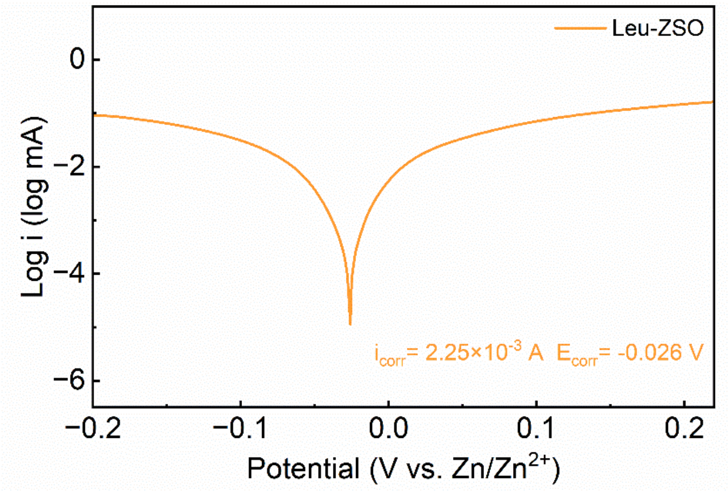

Figure S19. Tafel plots of the Zn//Ti cells in Leu-ZSO.

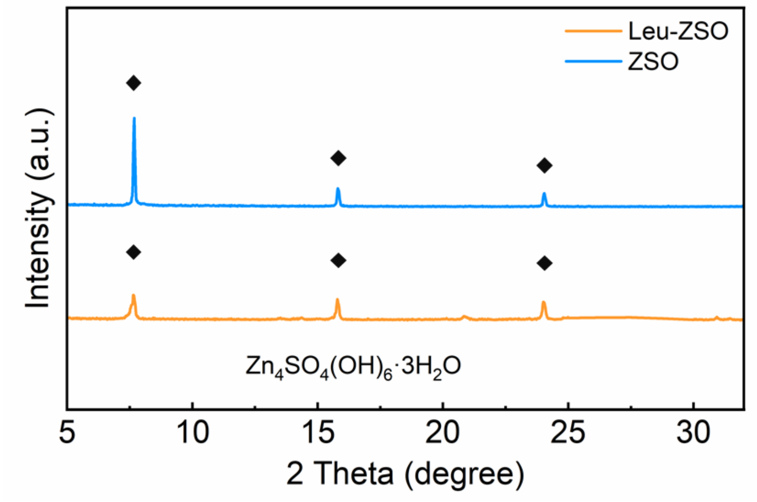

Figure S20. XRD patterns of Zn anodes after long cycling in Leu-ZSO and ZSO.

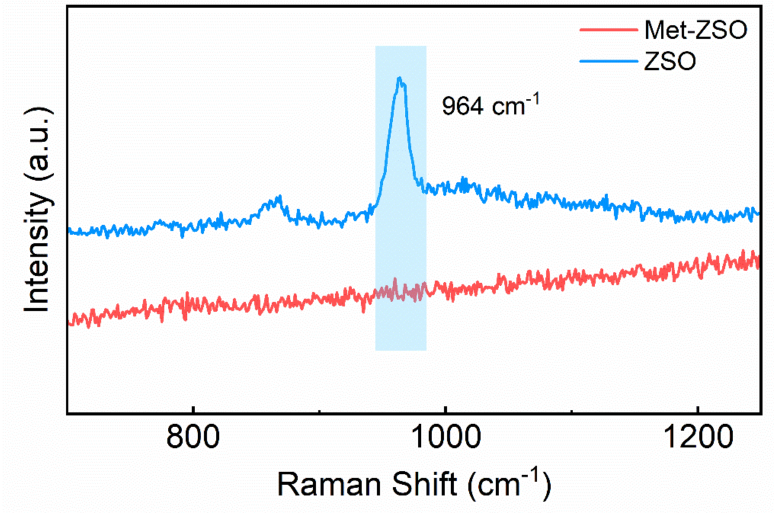

Figure S21. Raman spectra of cycling Zn anode in Met-ZSO and ZSO. The dominant peak at 964 cm^{-1} is chosen to show the Raman mapping.

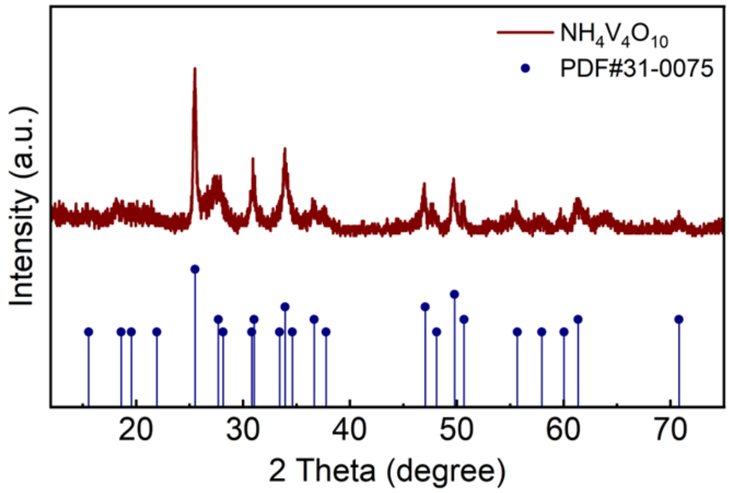

Figure S22. XRD patterns of NVO.

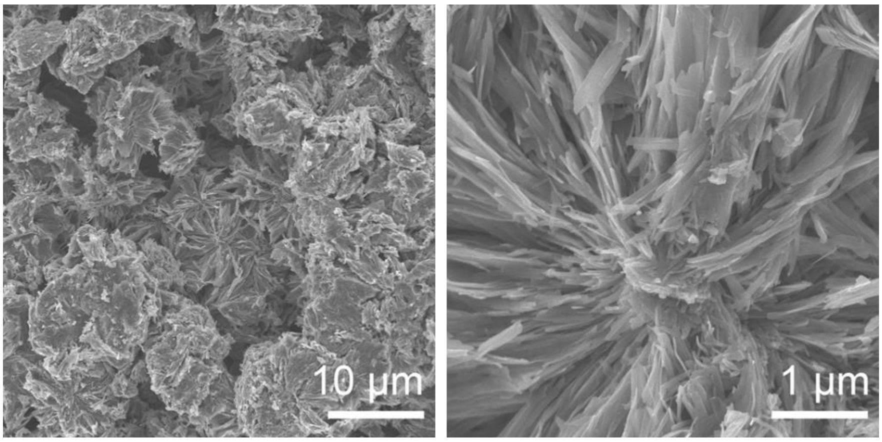

Figure S23. SEM images of NVO.

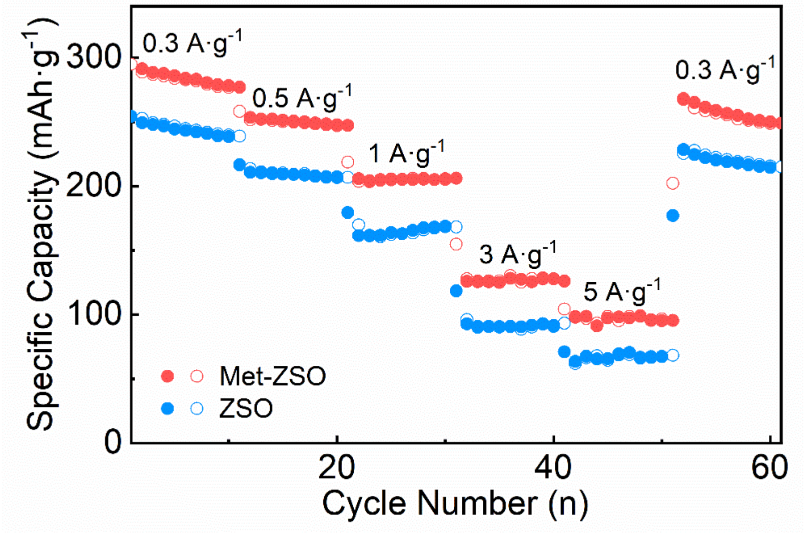

Figure S24. Rate performance of Zn//NVO full cells in Met-ZSO and ZSO under current density from 0.3 to 5 A g^{-1}.

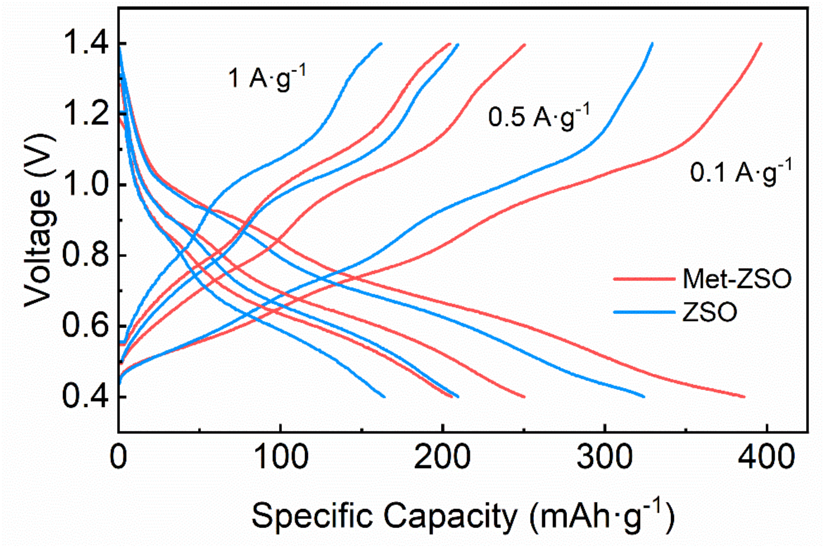

Figure S25. Voltage profiles of Zn // NVO full cells under 0.1, 0.5 and 1 A g^{-1} in two electrolytes.

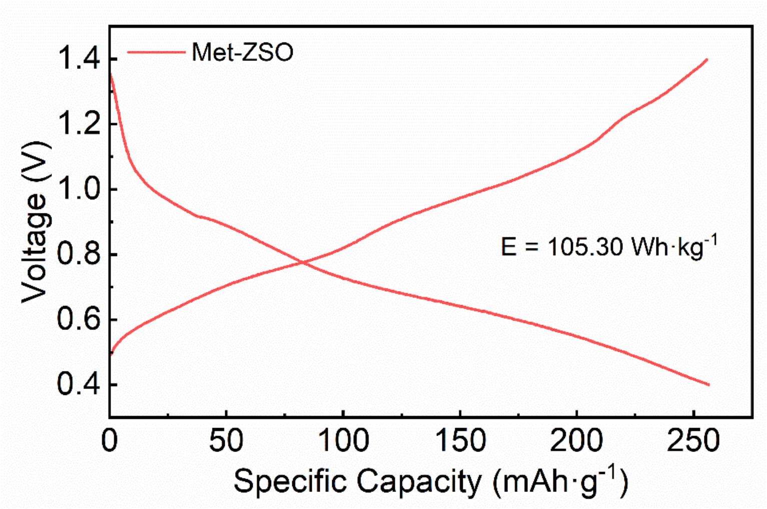

Figure S26. Voltage profile of Zn // NVO full cell under 0.3 A g^{-1} in Met-ZSO.

Table S1. Comparison of the electrochemical performance of Zn//Zn cells using aqueous electrolytes with additives in latest three years.

The kind of additive	Current density ($mA{\cdot}cm^{-2}$)	Capacity ($mA\ h{\cdot}cm^{-2}$)	Cycle Time (hour)	Ref. [a]
**This work**	**2**	**1**	**3750**	
	**5**	**1**	**1750**	
	**10**	**10**	**1580**	
	**20**	**5**	**620**	
	**30**	**30**	**110**	
β-cyclodextrin (β-CD)	4	2	1700	21
anionic sodium 3,3'-dithiodipropane sulfonate (SPS)	5	5	870	22

sodium anthraquinone-2-sulfonate (AQS)	0.5	0.5	2500	23
$CeCl_3$	2	1	2600	24
Sodium Tartrate (TA-Na)	5	2.5	430	25
Lanthanum Nitrate (La^{3+})	1	1	1220	26
L-carnitine (L-CN)	8.85	8.85	975	27
Xylitol	5	1	1000	28
tetramethylurea (TMU)	5	2.5	1600	29
Saccharin (Sac)	10	10	550	30

[a] The references are same as manuscript.

Table S2. Comparison of the electrochemical performance of Zn//Cu cells using aqueous electrolytes with additives in latest three years.

The kind of additive	Current Density ($mA\cdot cm^{-2}$)	Capacity ($mA\ h\cdot cm^{-2}$)	Cycle Number	Average CE (%)	Ref. [b]
**This work**	**2**	**1**	**3150**	**99.82**	
β-cyclodextrin (β-CD)	1	0.5	1700	99.56	21
sodium anthraquinone-2-sulfonate (AQS)	2	1	600	99.6	23
$CeCl_3$	2	1	300	99.8	24
Sodium Tartrate (TA-Na)	2	1	250	99.4	25
Monosodium Glutamate	2	1	1700	99.75	31

(MSG)					
Polyacrylamide (PAM)	1	1	1100	99.5	32

[b] The references are same as manuscript.

Table S3. Detailed data for calculating transference numbers.

	**ΔV (V)**	**R_{ct0} (Ω)**	**R_{cts} (Ω)**	**I_0 (A)**	**I_s (A)**
Met-ZSO	0.01	378.0	628.6	2.985e-5	1.757e-5
ZSO	0.01	165.8	304.2	2.766e-5	1.263e-5

References

[S1]Y. Fang, X. Xie, B. Zhang, Y. Chai, B. Lu, M. Liu, J. Zhou, S. Liang, *Adv. Funct. Mater.* **2021**, 32, 2109671.

[S2]S. J. Zhang, J. Hao, D. Luo, P. F. Zhang, B. Zhang, K. Davey, Z. Lin, S. Z. Qiao, *Adv. Energy Mater.* **2021**, 11.

[S3]M. Qiu, P. Sun, Y. Wang, L. Ma, C. Zhi, W. Mai, *Angew. Chem., Int. Ed.* **2022**, 61, e202210979.

[S4]C. Tian, J. Wang, R. Sun, T. Ali, H. Wang, B. B. Xie, Y. Zhong, Y. Hu, *Angew. Chem., Int. Ed.* **2023**, 62, e202310970.

[S5]Y. Zhou, G. Li, S. Feng, H. Qin, Q. Wang, F. Shen, P. Liu, Y. Huang, H. He, *Adv. Sci.* **2023**, 10, e2205874.

[S6]H. M. Yu, D. P. Chen, Q. Y. Li, C. S. Yan, Z. H. Jiang, L. J. Zhou, W. F. Wei, J. M. Ma, X. B. Ji, Y. J. Chen, L. B. Chen, *Adv. Energy Mater.* **2023**, 13.

[S7]J. Liu, B. Yuan, N. He, L. Dong, D. Chen, S. Zhong, Y. Ji, J. Han, C. Yang, Y. Liu,

W. He, *Energy Environ. Sci.* **2023**, 16, 1024.

[S8]Q. G. Jiang, Z. M. Ao, D. W. Chu, Q. Jiang, *J. Phys. Chem. C* **2012**, 116, 19321.

[S9]D. Wang, D. Lv, H. Liu, S. Zhang, C. Wang, C. Wang, J. Yang, Y. Qian, *Angew. Chem., Int. Ed.* **2022**, 61, e202212839.

[S10] M. Wang, J. Ma, Y. Meng, J. Sun, Y. Yuan, M. Chuai, N. Chen, Y. Xu, X. Zheng, Z. Li, W. Chen, *Angew. Chem., Int. Ed.* **2023**, 62, e202214966.

[S11] W. Yang, X. Du, J. Zhao, Z. Chen, J. Li, J. Xie, Y. Zhang, Z. Cui, Q. Kong, Z. Zhao, C. Wang, Q. Zhang, G. Cui, *Joule* **2020**, 4, 1557.

[S12] M. Qiu, P. Sun, K. Han, Z. Pang, J. Du, J. Li, J. Chen, Z. L. Wang, W. Mai, *Nat. Commun.* **2023**, 14, 601.

[S13] Q. Zhang, Y. Ma, Y. Lu, L. Li, F. Wan, K. Zhang, J. Chen, *Nat. Commun.* **2020**, 11, 4463.

[S14] X. Zhao, X. Zhang, N. Dong, M. Yan, F. Zhang, K. Mochizuki, H. Pan, *Small* **2022**, 18, e2200742.

[S15] H. Du, K. Wang, T. Sun, J. Shi, X. Zhou, W. Cai, Z. Tao, *Chem. Eng. J.* **2022**, 427.

[S16] Q. Meng, R. Zhao, P. Cao, Q. Bai, J. Tang, G. Liu, X. Zhou, J. Yang, *Chem. Eng. J.* **2022**, 447, 137471.

[S17] M. Wang, J. Ma, Y. Meng, J. Sun, Y. Yuan, M. Chuai, N. Chen, Y. Xu, X. Zheng, Z. Li, W. Chen, *Angew. Chem., Int. Ed.* **2022**, 135.
